# The construction of competency training mechanism model for tourism undergraduates based on grounded theory

**DOI:** 10.1371/journal.pone.0296683

**Published:** 2024-02-29

**Authors:** Guoxia Sun, Lan Zhao

**Affiliations:** 1 School of Tourism Culture, Tourism College of Changchun University, Changchun, 130607, China; 2 Faculty of Education, Northeast Normal University, Changchun, 130024, China; 3 Jilin Province Research Center for Cultural Tourism Education and Enterprise Development, Changchun, 130607, China; 4 Northeast Asia Research Center on Leisure Economics, Changchun 130607, China; Sichuan Agricultural University, CHINA

## Abstract

The motivation of this paper is to solve the problem of tourism majors’ lack of theoretical knowledge and professional ability by building a capacity training mechanism model based on grounded theory. The goal of the study is to optimize the ability training strategies of tourism undergraduates and improve their professional quality and competitiveness. The paper adopts the ability training model based on grounded theory, and combines with Back Propagation (BP) neural network for optimization and evaluation. By comparing the performance of different ability training mechanisms, this paper explores the best training strategies to provide support and guidance for the training of tourism undergraduates. Firstly, the employment background of the current market is studied and analyzed. By sorting out the relevant documents of grounded theory and combining with the current training strategies of tourism majors, the students’ personality characteristics and the basic principles of establishing models are integrated. The Back Propagation (BP) neural network is combined with the grounded theory. The data input of the student ability model is re-optimized. The undergraduate competency training mechanism model is constructed. The results show that when the number of iterations of the network model is 500.00, the evaluation accuracy of the competency training model based on BP neural network can reach 70.00%. At this time, the evaluation accuracy of competency training model based on content analysis method is only 55.00%. In addition, the results show that with the increase of model iterations, the recognition accuracy of the ability model based on grounded theory and the ability training mechanism of content analysis method is continuously improved. However, the ability evaluation model based on grounded theory has higher accuracy, and the accuracy of ability evaluation even exceeds 78.52% when the number of iterations of the network model is 600. Secondly, through the statistics and comparison of the grading results of students of different majors, it is found that the ability training mechanism based on grounded theory can improve the overall ability level of students more significantly. The research has important reference value for promoting the optimization and perfection of students’ training strategies.

## 1. Introduction

Nowadays, tourism has become one of the best ways for people to enjoy a leisurely life. With the establishment of the mobile network, the means of implementing tourism schemes are more and more diversified [1–3]. Meanwhile, with the development background of innovation strategy era, there are more and more business opportunities in tourism. However, the entrepreneurial ability of students majoring in the tourism is insufficient, so the ability building and training mechanism of students in tourism field needs to be improved. Using relevant theoretical knowledge to establish a competency training mechanism for students majoring in tourism as soon as possible can improve their innovation and integration ability, and provide some support for economic growth.

With the economic growth and the increase in college enrolment, the market supply of students exceeds the demand, and the market employment pressure is increasing. For students majoring in tourism, exploring entrepreneurial skills and developing skills will help improve their own competence and competitiveness, help them understand employment problems and provide employment support for them [4]. In addition, the training mechanism can promote the formation and improvement of basic theoretical knowledge through continuous data comparison and analysis. At present, grounded theory is characterized by clear steps, clear process and high operability [5]. Establishing a training mechanism model based on grounded theory can establish a theoretical-driven development model for students’ ability, meet the talent needs of the new stage of the tourism market, and further promote students’ personal career development needs. At present, the tourism industry is developing rapidly, so it is particularly important to strengthen the competence training of tourism undergraduates. Based on grounded theory, the competency training mechanism model of tourism undergraduates can be constructed from the following aspects: Firstly, strengthen theoretical knowledge education so that students can master the basic principles and operation methods of tourism business to form a deep understanding of the tourism market. Secondly, pay attention to the cultivation of practical operation ability, improve students’ proficiency in tourism business through practice and training to enhance their competence in practical work. Thirdly, strengthen the cultivation of innovative thinking and teamwork ability, and cultivate tourism professionals with innovative spirit and good communication skills to meet the ever-changing challenges of the tourism industry. Finally, pay attention to career planning and employment guidance, help students establish a correct concept of career development, and shape confident tourism talents who can adapt to market demand. Through a multi-faceted and comprehensive training system, the entrepreneurial ability of college students majoring in tourism can be effectively improved, and they can quickly adapt to the needs of the tourism market and contribute to economic growth.

On the basis of grounded theory, this paper analyzes the current situation of training strategies and competency training mechanism of students majoring in tourism, and further optimizes the way of data collection and collation by enhancing students’ professional awareness [6]. After analyzing the key factors of the training mechanism of tourism professional competence, the BP (Back Propagation) neural network model is used to re-optimize the input data of the model. Finally, the mechanism of cultivating ability of different models is compared through experiments. It is of great reference value to study and promote the improvement and optimization of tourism undergraduate students’ competence. The following criteria need to be considered when selecting the key factors of the tourism professional ability training mechanism. Firstly, the training mechanism should be targeted to meet the needs and development trends of the tourism industry. Secondly, the training mechanism should pay attention to the cultivation of practical ability, and improve students’ operational skills and management ability through field training and internship experience. In addition, students’ cross-cultural literacy and communication skills are also important factors, and they can adapt to the tourism environment with different cultural backgrounds and communicate and cooperate effectively with customers. Innovative thinking and problem-solving ability should also be taken into account to cultivate students’ competitiveness in the ever-changing tourism market. Finally, in order to ensure the cultivation of students’ comprehensive quality, educational institutions and enterprises should strengthen cooperation, provide opportunities for participation in practical cases and projects, and combine theoretical knowledge with practical application. The main contribution and innovation of the research is to construct a targeted, practice-oriented training mechanism for tourism professional ability, which pays attention to cross-cultural communication and problem-solving ability, and is helpful to improve the education and training system for tourism undergraduates.

## 2. Recent related work

### 2.1 Grounded theory

For the treatment of data saturation and the establishment of models, Finnerty et al. (2019) [7] conducted research on the application of grounded theory in psychological education of school students. The research explored the psychological education experience of school counsellor interns and the first generation of college students. Through the implementation of the core curriculum of school counselling related to career and university exploration, the results showed that grounded theory had important reference value for future psychological practice, training and research. Claramita et al. (2019) [8] studied the grounded theory in the design of undergraduate medical education on the basis of community education, and developed the basic learning framework based on community education. The results showed that the concrete practice of grounded theory could promote the further improvement of clinical skills, leadership and teamwork skills. Prikhidko et al. (2020) [9] conducted training and research on the emotional regulation effect of counsellors based on grounded theory. The results show that grounded theory plays an important role in emotional experience, emotional cognition and emotional self-management.

In addition, regarding the application of grounded theory in psychology and other fields, Charmaz et al. (2021) [10] studied the quality pursuit function of grounded theory in psychology, and compared and coded the data by combining the theory based on constructivism with grounded theory. The results show that the main forms and quality standards of grounded theory can promote the quality of model evaluation. Mousavi et al. (2021) [11] designed the self-leadership model of elite athletes based on grounded theory, and analyzed the qualitative data with a set of open, selective and theoretical coding methods. The reliability of the research results is verified by various methods and the calculation of consistency coefficient. The results show that elite athletes’ self-leadership includes four types: cognitive control, behavioral control, emotional control and management control. Hiltrop et al. (2021) [12] studied the rehabilitation process of long-term breast cancer survivors based on grounded theory, and collected data through written survey and semi-structured interview. The results emphasized the importance of survival nursing based on grounded theory and demand-oriented. Ahmady et al. (2022) [13] analyzed the situational problems of teaching and learning in clinical education based on grounded theory, and explained the teaching strategies in combination with discourse analysis. The results show that the clinical learning environment is a complex and multi-level social environment, and many social factors should be considered in the process of clinical teaching. Hilert et al. (2022) [14] conducted research on strategies to promote participation and empowerment through grounded theory, which conceptualized the systemic obstacles faced by volunteers and participants. By constructing the grounded theory model, the research can provide reference for promoting volunteer’s teaching practice.

To sum up, grounded theory is a research method to establish a substantive theoretical model, which has been applied in many fields at present. The research adopts grounded theory to establish the training mechanism model of students’ competence, and constructs a complete professional theory evaluation system to further improve the evaluation performance of the model.

### 2.2 Training strategy and competence training mechanism of tourism students

The current innovation mechanism and cultivation strategies of students are analyzed. For example, Siroj et al. (2021) [15] studied the improvement of innovative training in tourism education and investigated the current quality of tourism teaching in the higher education system by exploring the actual effects of innovative training in tourism education and the hotel industry. The results show that the optimization of teaching strategies for tourism majors can provide enough employment labour for the market. Trong et al. (2021) [16] conducted research on the training quality and training strategies of undergraduate students majoring in tourism in private colleges and universities, and used the survey samples of undergraduate students in the tourism field to explore the influence of online learning on learning quality. Through quantitative data analysis, the results show that most respondents’ satisfaction is higher than the neutral level when answering the factors that affect online learning. DOS (2022) [17] discussed the teaching challenges of undergraduate courses of tourism in Brazil. By studying and determining the main teaching methods and strategies of Brazilian teachers and adopting brand-new communication technologies and teaching schemes, the learning effect and employment rate of students have been greatly improved. Benuto et al. (2018) [18] sorted out the relevant materials on the cultivation of psychologists’ cultural ability, and analyzed the topics covered in the training courses. The results showed that the training of students’ cultural ability based on psychological science should be reconsidered. Callahan et al. (2019) [19] studied the training and education of vocational psychology for students and considered the key issues including the effectiveness of supervision, the differences and diversity of supervision. The results show that by improving the management of psychological training, students’ comprehensive competency level can be improved.

In addition to the comprehensive evaluation of students’ competency level, Cherdymova et al. (2019) [20] analyzed the social and psychological factors that promoted and hindered the formation of students’ eco-professional consciousness, and created a teaching complex through practical modelling. The research can provide reference for the cultivation of students’ ecological professional consciousness and competence. Ru et al. (2020) [21] optimized the training mode of human anatomy teachers with post competence as the core. The results show that the construction of the four-in-one training mode, which includes talent leadership, humanistic spirit cultivation, teaching ability and scientific research quality, can reserve an important force for discipline development. Chang et al. (2020) [22] studied the evaluation and promotion system of employees from the perspective of competency, taking the employees in the workshop of the company as the research object. This paper summarizes the development of competency theory in China and other countries, the definition of competency concept, and the overview of the competency model. The results show that training employees based on competency model can improve their self-ability and promote the high-quality development of enterprises. Tran et al. (2021) [23] adopted the competency training mechanism to optimize the education and training strategies of senior high school teachers in the delta region, and managed teachers’ ability in the delta region according to the ability method. The results show that by providing teaching and training activities for gifted senior high school teachers, teachers’ education and management level can be further improved. Dou et al. (2023) [24] developed an evaluation and prediction system to evaluate the development of advanced manufacturing industry in cities. The research adopted the method of establishing evaluation index system and prediction model for evaluation and prediction. Li et al. (2022) [25] made a meta-analysis on the application of highly realistic simulation in undergraduate nursing education. It was found that highly realistic simulation played a positive role in improving the skill level of nursing students. Pong (2021) [26] conducted a longitudinal mixed study on cultivating college students’ spiritual well-being in holistic education. The results showed that holistic education could promote the spiritual well-being of college students. Barnes et al. (2021) [27] discussed the student-centered free travel mode through the case study of Johannesburg, and provided relevant evidence to support it. Li et al. (2022) [28] analyzed the policy discourse of talent cultivation in China’s establishment of a world-class university. It was found that talent training was of great significance for China to establish a world-class university. Zhang (2021) [29] reformed the practical teaching system for cultivating applied undergraduates in local universities. The research results suggested that the practice teaching reform was helpful to the cultivation of applied undergraduates. Du et al. (2021) [30] investigated the undergraduate education of IoT engineering in China. It was found that there were some problems in undergraduate education of IoT engineering, which needed to be improved.

In addition, Milawaty et al. (2023) [31] identified the need for students in tourism destination research projects to offer English public speaking courses. Ghebreyessus et al. (2021) [32] studied the methods of cultivating success through undergraduate research experience in black colleges and universities with a long history. The results reflected that undergraduate research experience had a positive impact on students’ success. Kumar et al. (2022) [33] put forward effective undergraduate training principles to improve undergraduate students’ relevant teaching strategies. Wang et al. (2021) [34] studied the diversified talent training mechanism of early childhood physical education based on the concept of whole-process practice. They found that early children’s physical education oriented to children’s mental health and intelligent teaching was helpful to cultivating diversified talents. Scheibenzuber et al. (2021) [35] designed a problem-based undergraduate online course to improve students’ awareness of fake news literacy. Zhou (2021) [36] studied the problems and countermeasures of cultivating the innovation and entrepreneurship ability of adult college students in the Internet age. The research put forward specific countermeasures for the cultivation of innovation and entrepreneurship ability of adult college students. Gardner et al. (2021) [37] found that the undergraduate education of protection and environment in British universities lacked interdisciplinary. The research adopted the methods of literature review and in-depth interview, and emphasized the importance of introducing interdisciplinary teaching into undergraduate education of protection and environment. Alkhaifi et al. (2022) [38] systematically reviewed the undergraduate medical education and training based on visual arts. The research adopted the method of systematic review, summarized the relevant literature, and discussed the influence and effect of visual art on undergraduate medical education.

The analysis of the above research literature suggests that the current research on students’ innovation mechanism and training strategies involves different aspects in the field of education. In the field of tourism education, optimizing teaching strategies can provide sufficient employment labor for the market. In addition, the research also shows that the comprehensive quality level of students can be improved by enhancing the cultural ability cultivated by psychology and the management ability of vocational psychology training. In other fields, such as ecological specialty, medical education and engineering training, different teaching modes and strategies can also achieve remarkable results. Summing up the above research table, it is clear that all fields are seeking innovation and improvement in student training to improve students’ ability and meet market demand. Therefore, it is very important to constantly study and improve educational strategies for cultivating students’ comprehensive quality and professional ability.

## 3. Construction of content system of student competency training mechanism model based on grounded theory

### 3.1 Students’ personality characteristics and the principles of competence cultivation

With the passage of time, the methods of grounded theoretical research are constantly optimized and improved. The basic method of grounded theory is to analyze and deepen the collected data by means of classification and summary, and develop a set of standard theories. In this process, data acquisition and collection are the core of the research method. Firstly, the collected data are classified, subdivided and conceptually represented. Then, the concept information is merged and improved. In addition, the cultivation of students majoring in tourism is crucial to the future development of the industry [39,40]. In the research on the cultivation of tourism majors, it is very important to analyze students’ personality characteristics. It is necessary to deeply understand students’ hobbies, learning motivation, communication skills and other factors in order to provide them with more personalized education and training resources. The research understands students’ basic information, hobbies and career planning through in-depth interviews and exchanges. Secondly, in the practice course or internship, the students’ performance and communicate with the enterprise tutor are observed to get feedback on the students’ actual working ability. One-on-one interviews are conducted to deeply understand students’ voices and needs, which is helpful for further analysis and summary and provides scientific basis for the training of tourism professionals. In order to obtain more comprehensive data, the research team also conducted in-depth interviews with individuals or groups. In these interviews, researchers have face-to-face communication with selected students to gain a deeper understanding of their views, experiences and needs on the capacity training mechanism. These interviews provide qualitative data for the research and enrich the dimension and depth of the research results. In addition, the research team also refers to the relevant data of UCI Machine Learning Repository, an open experimental dataset. This online machine learning repository provides a number of datasets for research, including travel-related datasets, such as comments on tourist attractions and travel behaviors. These data provide valuable information about the theoretical framework, best practices and case studies of students’ ability training mechanism. By comprehensively analyzing these data, the research team can fully understand the ability training needs of students majoring in tourism and music, and provide strong support for building an effective ability training mechanism model. The personality characteristics of students and the principles of establishing a competency training mechanism are analyzed.

However, at present, there is little literature about tourism talent training programs, and most colleges and universities rely on the traditional tourism talent training mode. Therefore, for a systematic and comprehensive analysis of the current situation of tourism training programs, it is necessary to establish a model to evaluate the competition of tourism sector according to the competition model and the characteristics of major competitors. For the key factors of tourism professional competence training mechanism, the results of factor analysis are shown in Table 1.

**Table 1:** Key factors of tourism professional competence training mechanism.

Evaluation index	Inducement and classification of elements
Communication and interpersonal skills	Expression ability
Master the basic theoretical knowledge of tourism major	Professional knowledge and ability
Mastery of English or other foreign languages	Foreign language ability
Ability to use computer software for data processing	Information technology ability
The ability to keep learning and quickly master new knowledge	Continuous learning ability
Ability to work as a team to solve practical problems	Team cooperation ability

Table 1 lists the key factors of tourism professional competence training mechanism and their induction and classification. Among them, the ability of communication and interpersonal communication is an important factor, which includes the ability of expression; Mastering the basic theoretical knowledge of tourism specialty involves professional knowledge and ability; Proficiency in English or other foreign languages requires foreign language ability; Knowing how to use computer software for data processing requires information technology ability; Maintaining learning ability and quickly mastering new knowledge reflect continuous learning ability; Teamwork to solve practical problems requires teamwork ability. These factors and elements are all important parts of the training mechanism of tourism professional ability. Through the cultivation and promotion of these factors, students majoring in tourism can better adapt to and cope with the challenges and needs in their work. Meanwhile, these indicators also provide a basis for evaluating students’ performance and progress in the process of tourism professional ability training. In order to ensure the scientificity of the indicators in Table 1, the relevant research literature of Mínguez et al. (2021) [41] is studied, and the reliability of the indicators is verified. According to the key factors of tourism professional ability training in Table 1, these factors can be deeply analyzed through grounded theory. First, it is necessary to systematically collect all kinds of tourism professional education resources, practical courses, teacher-student feedback and other data. On this basis, using classification and induction methods, the collected information corresponds to the evaluation indicators in Table 1. Next, through comparative analysis, people can further find the importance and advantages of each factor in the training process, as well as possible shortcomings. The results of factor analysis show that communication and interpersonal skills, basic theoretical knowledge of tourism, English or other foreign language skills are the key to improve students’ comprehensive quality. In addition, information technology ability, continuous learning ability and teamwork ability are equally important. In view of these key factors, the education department needs to adjust and optimize the training program to promote the all-round and balanced development of tourism majors. Meanwhile, enterprises are encouraged to participate in students’ practical projects to increase students’ sensitivity and adaptability to the market. In a word, the results of factor analysis provide strong support for formulating more scientific and effective training strategies for tourism professionals.

### 3.2 Optimization of competency model by BP neural network

After the students’ competency model is built, the input data of the model is re-optimized by BP neural network algorithm with back propagation of errors. Assuming that the input of the sample is *A*_*k*_ = (*a*_1_, *a*_2_, *a*_3_,…,*a*_*m*_), the input value *S*_*j*_ of the hidden layer of the neural network is calculated, and the equation is as follows:

$$S_{j}=\sum_{i=1}^{m}\;w_{ij}a_{i}-\theta_{j}$$ (1)

*S*_*j*_ is the input value of the hidden layer, *i* is the serial number of the input unit, and *j* represents the serial number of the hidden layer unit. *a*_*i*_ is the ith input value, *w*_*ij*_ is the connection weight between the input layer and the hidden layer, and *θ*_*j*_ is the threshold of neurons in the hidden layer. The activation function value of the neural network is calculated, and the equation is as follows:

$$b_{j}=f(s_{j})=\frac{1}{1+e^{-s_{j}}}$$ (2)

*b*_*j*_ represents the output value of the nonlinear activation function, and *s*_*j*_ represents the input value of the hidden layer. The input value *l*_*t*_ of the output layer is calculated, and the equation is as follows:

$$l_{t}=\sum_{j=1}^{p}\;v_{jt}b_{j}-r_{t}$$ (3)

*r*_*t*_ represents the threshold of neurons in the output layer, *v*_*jt*_ represents the connection weight between the hidden layer and the output layer, and *t* represents the serial number of the output unit. After the forward transmission process of the model is completed, the error of the model is corrected through the backward transmission of data. The error correction mode of the output layer is as follows:

$$d_{t}=(y_{t}-c_{t})\cdot c_{t}(l-c_{t})$$ (4)

*c*_*t*_ represents the actual output of the t-th neuron, (*y*_*t*_−*c*_*t*_) is the absolute error between the expected output and the actual value of the sample, and *c*_*t*_(*l*−*c*_*t*_) represents the first derivative of the activation function of the output layer. The connection weight correction *v*_*jt*_(*N*+1) between the output layer and the hidden layer of the model is calculated, and the equation is as follows:

$$v_{jt}(N+1)=v_{jt}(N)+a\cdot d_{t}\cdot b_{j}$$ (5)

*a* represents the learning factor, and *v*_*jt*_(*N*) represents the connection weight correction amount between different hidden layers of the model. the correction *e*_*j*_ in the hidden layer is calculated, and the equation is shown as follows:

$$e_{j}=(\sum_{t=1}^{q}\;d_{t}\cdot v_{ij})b_{j}(1-b_{j})$$ (6)

*b*_*j*_(1−*b*_*j*_) represents the first-order differential of the activation function of the hidden layer. The connection weight between the hidden layer and the model output layer is calculated, the equation is as follows:

$$w_{it}(N+1)=w_{it}(N)+\beta \cdot e_{j}\cdot a_{i}$$ (7)

*w*_*it*_(*N*+1) represents the connection weight between the hidden layer and the model output layer, and *β* is the momentum factor. The established model of student competency optimization is improved.

In the established student ability optimization model, BP neural network algorithm is used to re-optimize the input data. In the research, the deployment and application process of statistical model is as follows: First, students’ grades and characteristic data are collected as input samples, and these data are input into BP neural network. The output value is obtained by calculating the input value of the hidden layer and the activation function (Eq 2). Then, the input value of the output layer is calculated according to Eq 3. After the forward propagation of the model is completed, the error of the model is corrected by the backward propagation of data. Firstly, the error correction of the output layer is calculated (Eq 4), and then the connection weight correction between the hidden layer and the output layer is calculated (Eq 5). Subsequently, the correction of hidden layer is calculated according to Eq 6, and the connection weight between model output layer and hidden layer is calculated according to Eq 7. Through repeated iterative training, the weights and thresholds are gradually adjusted to appropriate values, so that the model can get better prediction results. In practical research, this model can be applied to analyze students’ ability level and provide targeted programs for teaching management and counselling. Online education platform can recommend suitable curriculum resources and learning paths for students according to the results predicted by the model to improve their learning effect.

### 3.3 Experimental environment setting and research

In order to verify the performance of the training mechanism model, the training mechanisms of different models are compared through experiments. The experimental hardware configuration is: Central processing unit (CPU) is Intel(R)Core (TM)i7-7700, the main frequency is 2.50GHz, the memory is 16GB(Gigabyte), the storage hard disk bit is 5.2T, the video memory is 8GB, and the operating system is Windows10 with 64 bits. After the selected experimental competency indicators are merged and revised, the index characteristics of data elements are extracted. According to the ratio of 8:2, the dataset collected by the research is divided into research training set and test set. According to the competency model, students’ professional skills and training mechanism can be identified quickly and effectively. In order to verify the performance of the training mechanism model, it is necessary to encode the data first. For the selected experimental competency index, the category data can be converted into numerical data by One-Hot Encoding or Label Encoding, which is convenient for subsequent calculation and analysis. Next, according to the principle of grounded theory, the index features are extracted by induction, coding and comparison of various data elements. When establishing the theory, the research should follow the following steps: 1) Open coding: analyze and classify the original data in detail and extract key concepts; 2) Axial coding: explore the relationship between different concepts and form a higher-level abstract concept; 3) Selective coding: select the core category from it, and make in-depth analysis around the core category to establish a complete and well-founded theoretical system. In this way, the experimental results can be combined with theoretical knowledge to provide strong support for the competency training mechanism model of tourism undergraduates.

In addition, in the process of establishing the model of students’ competency training mechanism, the data is pre-processed by BP neural network structure, and the model can be retrained and adjusted according to the needs of subsequent performance evaluation. In order to compare the performance of the model, this paper compares the competency training model based on grounded theory with the competency training mechanism based on content analysis. Meanwhile, in order to highlight the professional theoretical ability of undergraduate students majoring in tourism, this paper analyzes and compares the competency indexes of students majoring in tourism and music. In the process of establishing the model of students’ ability training mechanism, the data is pre-processed by BP neural network structure, and the model can be retrained and adjusted according to the needs of subsequent performance evaluation. In order to compare the performance of the model, this paper compares the ability training model based on grounded theory with the ability training mechanism based on content analysis. Meanwhile, in order to highlight the professional theoretical ability of undergraduate students majoring in tourism, this paper analyzes and compares the ability indexes of tourism specialty. The setting parameters of Artificial Neural Network-Back Propagation (ANN-BP) model are explained in detail as follows: ANN-BP model includes input layer, hidden layer and output layer, among which the number of nodes and iteration times of hidden layer are important parameters to be set. In terms of network structure, the BP neural network model used in this paper includes input layer, hidden layer and output layer. The input layer receives students’ grades and characteristics as input samples, the hidden layer is responsible for extracting and representing the high-order characteristics of data, and the output layer outputs the prediction results of students’ ability level. Setting the number of nodes and iterations of the hidden layer needs to be reasonably selected for specific problems and datasets. The determination of the number of hidden layer nodes is usually based on the analysis of the characteristics and complexity of training samples. If the sample features are complex, the number of nodes in the hidden layer can be appropriately increased to improve the fitting ability and performance effect of the model. The number of iterations can be adjusted by cross-validation and other methods to find a suitable training number, which can reduce the training error and avoid the problem of over-fitting. In this paper, the number of hidden layer nodes is set to 20, and the number of iterations is set to 1000. After experimental adjustment, it is found that this network structure can achieve a better model representation effect, that is, it can properly handle the randomness and complexity of data while maintaining the stability and reliability of the model.

During the operation of the algorithm, due to the randomness of the structure of BP neural network, even if the same parameter settings are adopted, the training results may be different. This is because the randomness of the initial connection weight and threshold leads to the network may fall into different local minima in the process of reverse propagation. Therefore, when using ANN-BP model to train data, it is necessary to run the results several times and take the average value to reduce the impact of randomness. Compared with other models, the ability training model based on BP neural network proposed in this paper has the following advantages. First of all, the model can be retrained and adjusted according to the actual needs to adapt to the changes in subsequent performance evaluation. Secondly, by comparing with the model based on content analysis, this paper proves the effectiveness of the model based on BP neural network in students’ ability training. In addition, this paper also highlights the professional theoretical ability of undergraduate students majoring in tourism by analyzing the ability indicators of students majoring in tourism and music. In a word, the ability training model based on BP neural network has the advantages of flexibility, effectiveness and pertinence, and can provide students with personalized teaching management and counseling programs. Although the randomness of the algorithm may lead to the difference of training results, a more stable and reliable model performance can be obtained by running for many times and taking the average value to reduce the influence of randomness. Based on the grounded theory, the algorithm of establishing the competency training mechanism model of tourism undergraduates is carried out by systematically analyzing and coding related research texts. The algorithm first creates an initial coding list based on the research questions and objectives, and then iteratively reads and analyzes the research text, identifies related concepts and assigns appropriate coding. Next, similar codes are compared and classified into different categories. Through constant comparison and revision, the codes and categories are further refined and revised. Finally, the algorithm outputs codes representing concepts, categories and relationships. Such a process can help researchers deeply understand and understand the key elements and relationships of the competency training mechanism model for tourism undergraduates. The pseudo-code flow of the algorithm for establishing the competency training mechanism model of tourism undergraduates based on grounded theory is shown in Table 2.

**Table 2:** Pseudo-code flow of algorithm for establishing competency training mechanism model of tourism undergraduates based on grounded theory.

1	**Algorithm: Grounded Theory**
2	**Input: Research texts related to competency training mechanism for tourism undergraduates** ***P* = {*p***_**1**_ **, *p***_**2**_ **,…,*p***_***n***_ **}**
3	**Output: Codes representing concepts, categories, and relationships**
4	**Parameters:** $b_{0},b_{k},H,\mu ,\lambda ,C_{th},V_{s_{n}}$
5	Create an initial list of codes based on the research questions and objectives
6	Iteratively read and analyze the research texts
7	Identify relevant concepts and assign appropriate codes
8	**For** *j* = 0: *k*−1 do
9	$Grid(S_{x},b_{1})=\{p_{i}=(x_{i},y_{i},z_{i})\}$
10	$Grid(S_{x},b_{1})=\{p_{i}=(x_{i},y_{i},z_{i}+H)\}$
11	Order the points in the grid Grid (*S*_*x*_, b_1_) at height h
12	Compare and group similar codes into categories
13	**IF** $p_{1}^{2}>C_{w}$
14	Return p_*i*_
15	**Else**
16	**While** $\Delta z=p_{j+1}-p_{i}^{2}<V_{ta}$
17	Refine and revise the codes and categories through constant comparison;
18	**End while**
19	**End IF**
20	**End For**
21	**End For**
22	**Return *p***_***g***_

## 4. Results and discussion

### 4.1 Performance analysis of different models for students’ competency recognition

**Fig 1:**
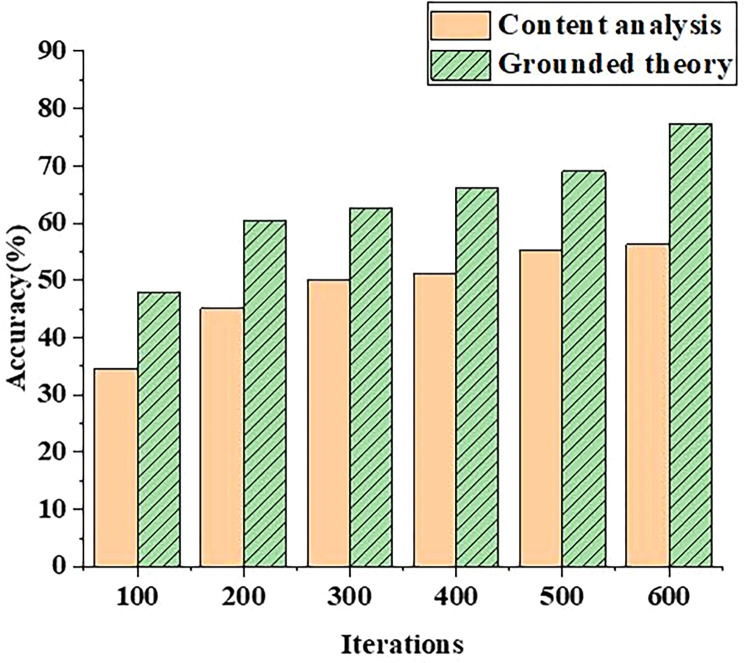
Comparison of competency recognition accuracy between grounded theory and content analysis method.

**Fig 2:**
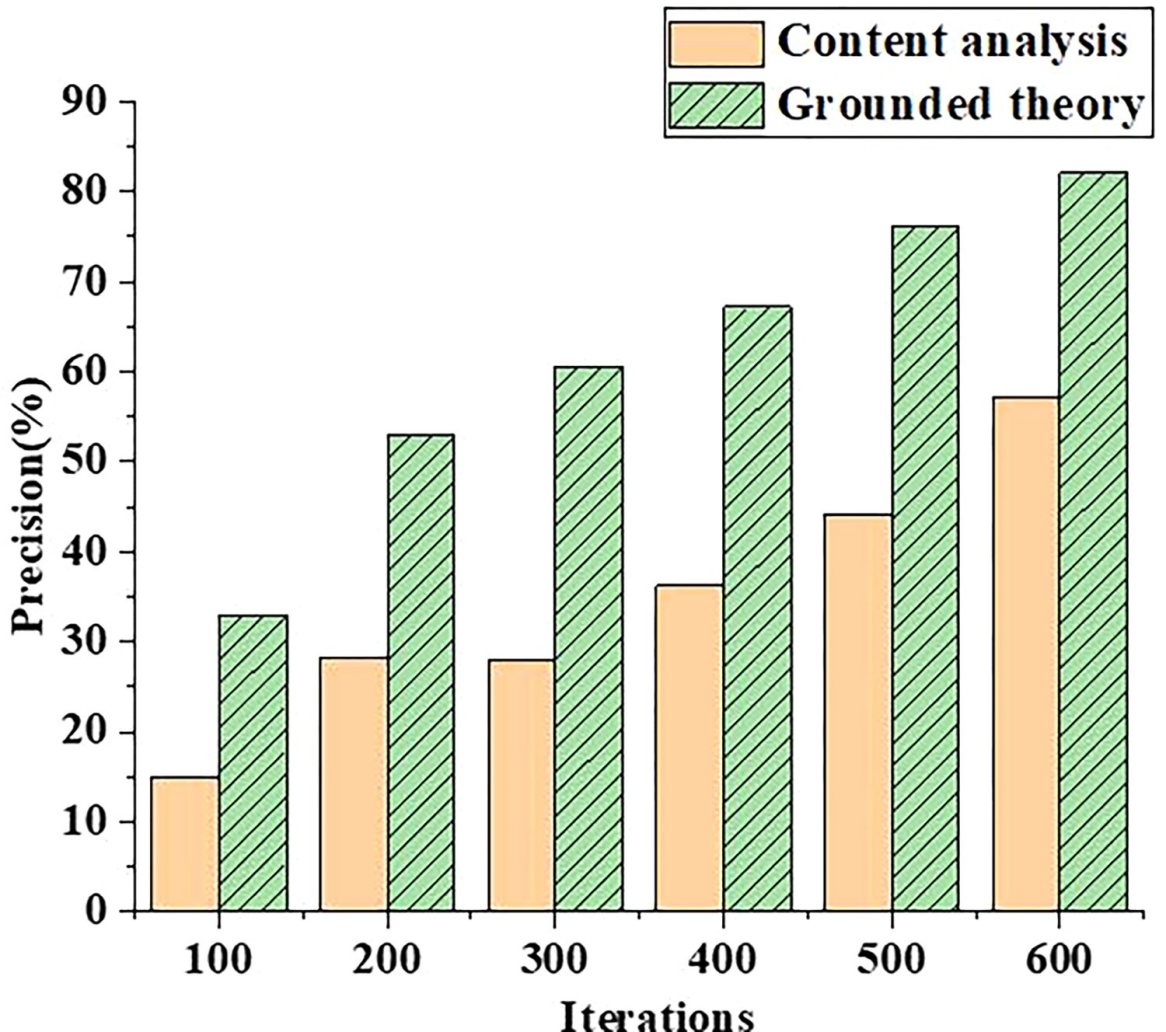
Comparison of the accuracy of competency recognition between grounded theory and content analysis method.

In order to compare the performance of the competency training model based on grounded theory, the competency training mechanism model based on grounded theory is compared with the competency training mechanism based on content analysis. The data from the accuracy and precision of model competency identification are analyzed, and the results are shown in Figs 1 and 2.

From Fig 1, with the increase of iteration times of the model, the recognition accuracy of competency model based on grounded theory and content analysis method is constantly improving. When the number of model iterations is 100.00, the accurate recognition value of competency training mechanism based on content analysis method is only 35.00%, while that of competency model based on grounded theory can reach 50.00%. In addition, when the number of iterations of the network model is 500.00, the accurate value of the grounded theory can reach 70.00%. When the number of iterations of the model increases to 600.00, the accuracy of competency assessment based on grounded theory proposed in this paper can reach more than 78.52%. Therefore, the competency evaluation model based on grounded theory is more accurate. Besides, it can be seen that grounded theory is qualitative research through constructivism, while content analysis is quantitative research through positivism. In terms of operation process, the content analysis method emphasizes the standardization and systematization of course content, while the grounded theory focuses on induction and verification. The results show that the recognition accuracy of the model integrated into the BP neural network is higher than that of the recognition model based on content analysis.

In Fig 2, with the increase of iteration times of the model, the accuracy of capability evaluation of different competency mechanism models is constantly improving. In 100.00 iterations of the model, the prediction accuracy of competency training mechanism based on content analysis method is 15.34%, while that based on grounded theory is 34.23%. The results show that the competency training mechanism based on grounded theory can improve the overall ability level of students. Additionally, the research on the theory is mainly diversified data analysis problems, and the coding of data enables the data model to be continuously deduced and inducted. The effectiveness of the established competency evaluation model is tested by analyzing the recognition accuracy and precision of different models. The test results show that the selection of different measurement factors in the model is appropriate, and an appropriate and accurate evaluation model can be established according to the factor weights.

### 4.2 Statistics and comparison of grading results of students of different majors

**Fig 3:**
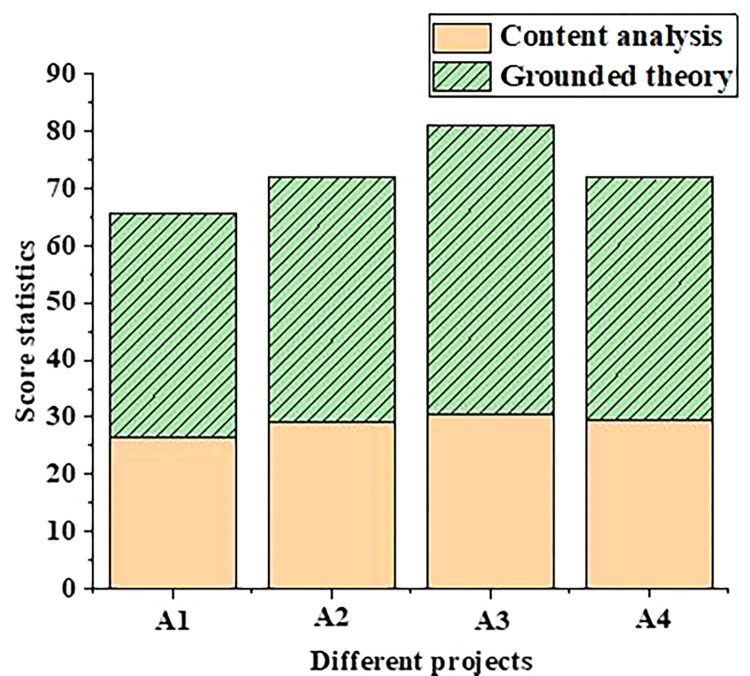
Statistics of scores of music majors based on different competency training mechanisms (A1: Practical ability; A2: Innovative ability; A3: Cooperation ability; A4: Lifelong learning ability).

**Fig 4:**
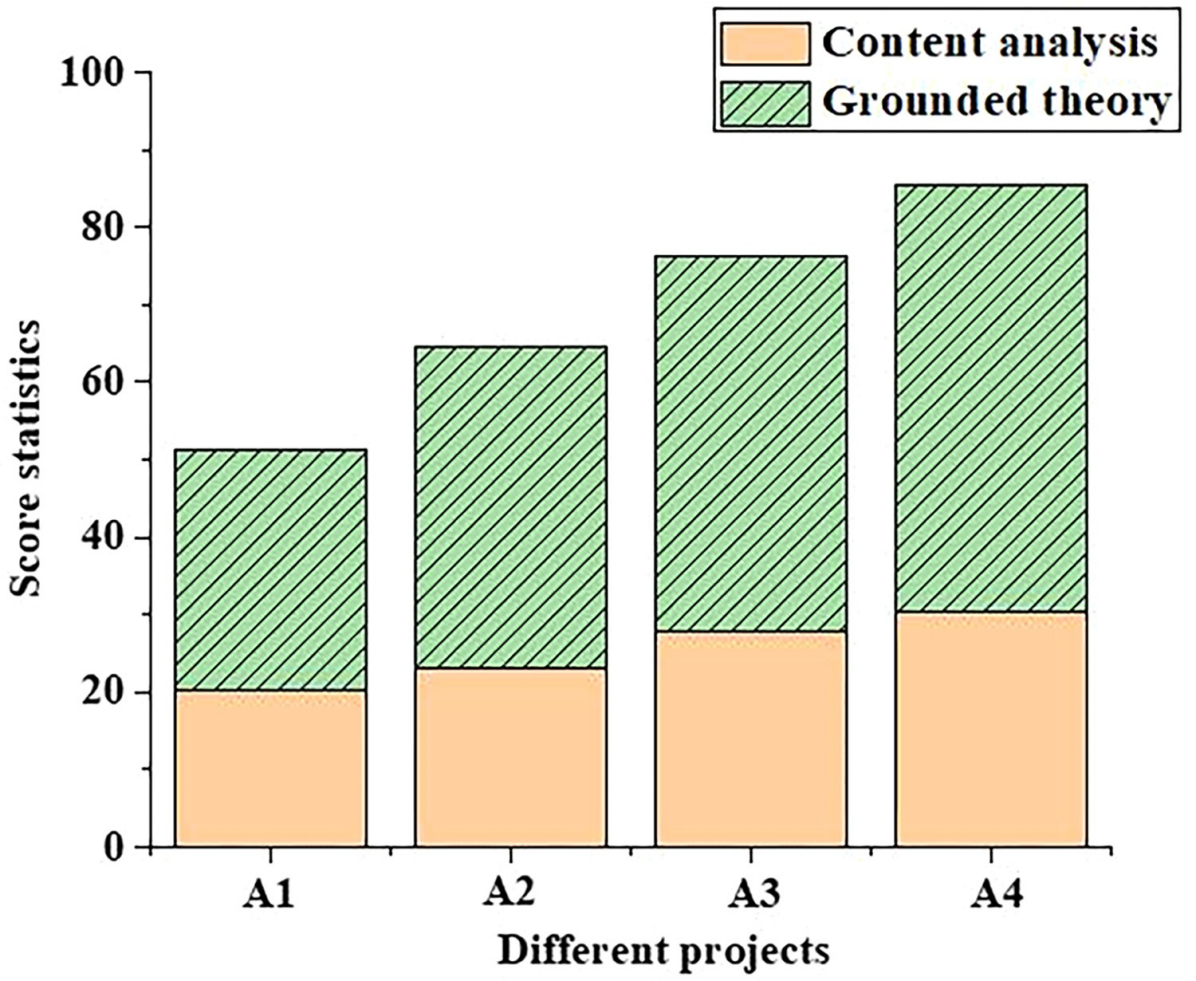
Statistics of the grading results of tourism majors based on different competency training mechanisms (A1: Practical ability; A2: Innovative ability; A3: Cooperation ability; A4: Lifelong learning ability).

In addition, in order to compare the differences in professional theoretical knowledge between undergraduate students majoring in tourism and those of other majors, the scores of different competencies of the undergraduate students majoring in tourism are input into the model and compared with those of the undergraduate students majoring in music. The scores of model ability of music majors and tourism majors are shown in Figs 3 and 4, respectively.

Fig 3 shows that the content analysis and grounded theory are used to compare the scoring results of different items of music majors, and the scoring results of the two models are different. After the competency training mechanism is established based on the content analysis method, the scores of students’ innovative ability and practical ability are 29.00 and 31.00 respectively, while the scores of students’ practical ability are only 26.00. By adopting the competency model framework based on grounded theory, the students’ score in practical ability is 39 points, and the students’ score in cooperation ability is the highest, with 50 points. Therefore, the model performance of competency training mechanism based on grounded theory is superior.

The same way is used to analyze the different competency training abilities of tourism majors. From Fig 4, the content analysis method is used to establish the competency training mechanism, and the practical ability of tourism majors is only scored 20 by this mechanism. Under the same conditions, grounded theory is used to cultivate and model competency, and grounded theory scores 31 points for students’ practical ability. In addition, by analyzing the different abilities of the competency model established by grounded theory, the lifelong learning ability score of tourism majors is the highest, with 55 points. Therefore, the competency model based on grounded theory can be popularized to further improve students’ theoretical and cultural level.

### 4.3 Comparison of project scores of different competency training mechanisms

**Fig 5:**
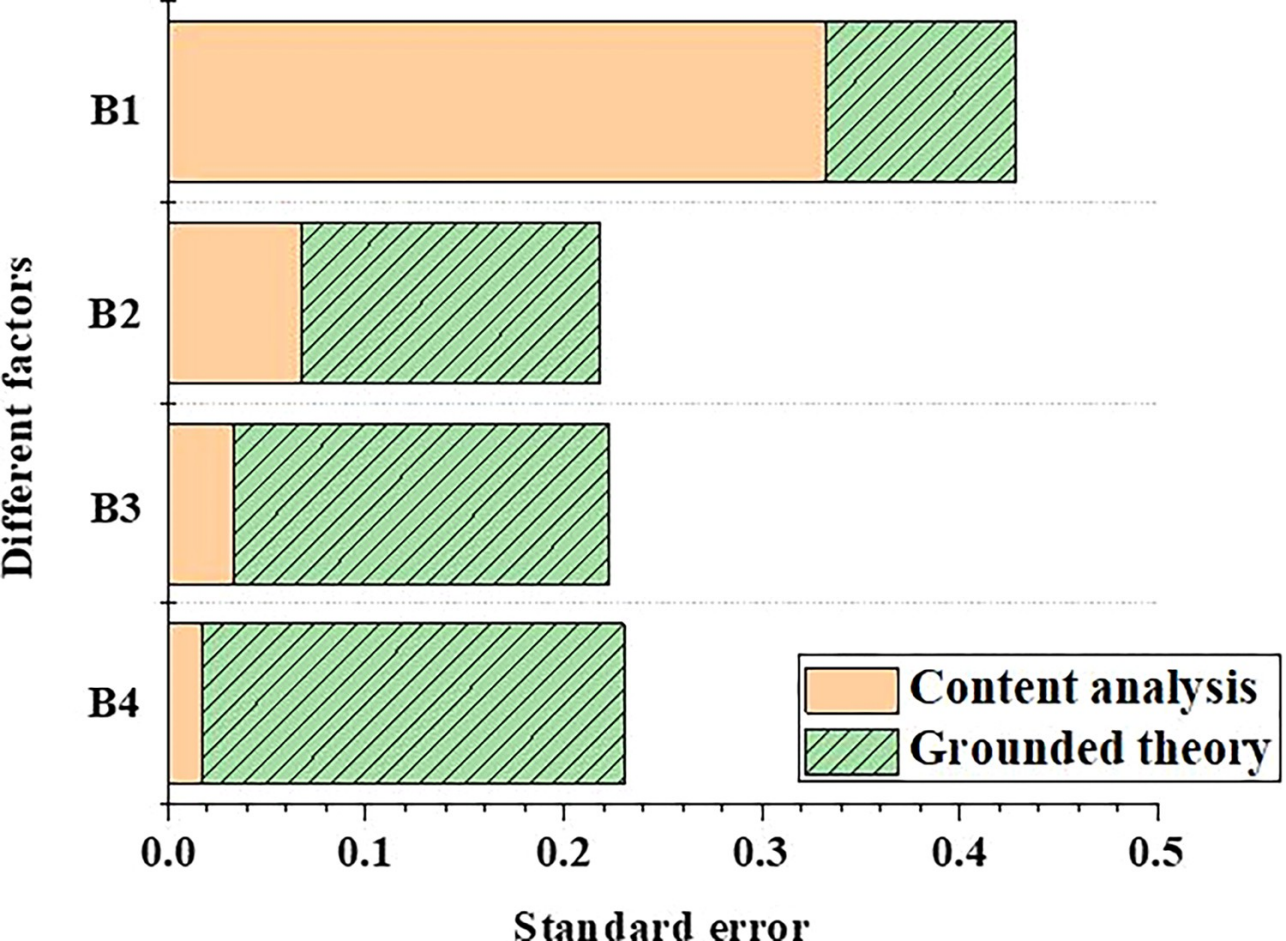
Comparison of standard errors of training programs for tourism majors with different competency training mechanisms (B1: Logical thinking ability; B2: Communication skills; B3: Foresight consciousness; B4: Information technology capability).

**Fig 6:**
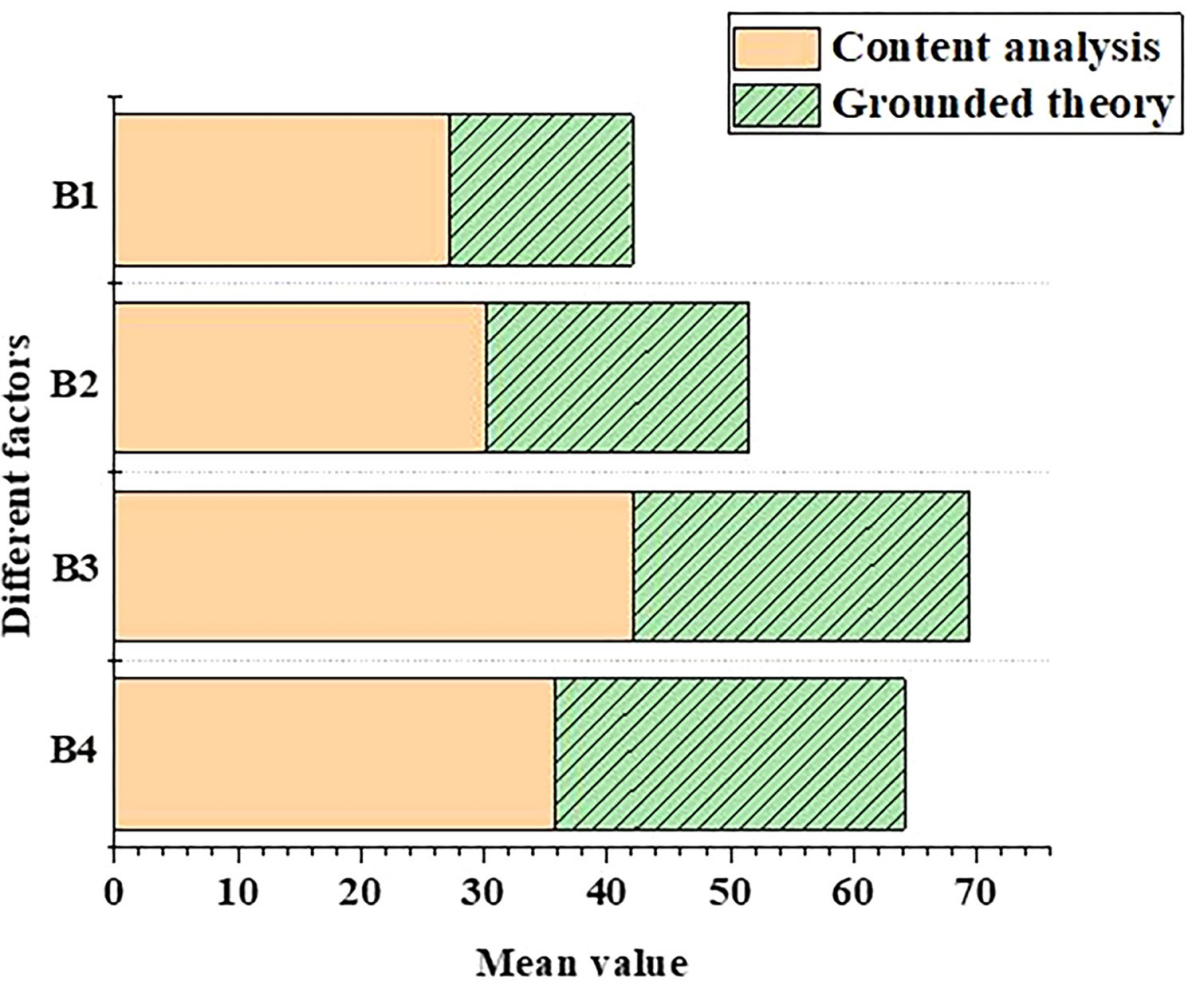
Comparison of average scores of training programs for tourism majors with different competency training mechanisms (B1: Logical thinking ability; B2: Communication skills; B3: Foresight consciousness; B4: Information technology capability).

In order to compare the difference of scores of different training programs in the competency training mechanism, the scoring data of tourism major students’ training programs are input into the model, and the data are processed and analyzed. The results of standard error and average score of tourism students’ training programs are shown in Figs 5 and 6, respectively.

In Fig 5, different training mechanism models have different evaluation levels for tourism majors’ competence. Among them, the training mechanism based on content analysis method has the smallest scoring error for students’ information technology ability, only 0.01. In addition, the model has the largest scoring error of 0.33 for students’ logical thinking ability. Meanwhile, the competency training mechanism model based on grounded theory is established. The scoring error of this model for students’ logical thinking ability is only 0.09, while the scoring error for students’ information technology ability is 0.21. Therefore, combining the advantages of content analysis method, the competency mechanism established by grounded theory can be optimized to further reduce the prediction error of the model.

From Fig 6, the training mechanism based on content analysis method has a big difference in evaluation scores of students’ different abilities, while the training mechanism based on grounded theory has a small difference in evaluation scores of students’ different abilities. In terms of students ’ logical thinking ability, the score of the training mechanism based on content analysis is 27.00, while the score of the training mechanism model based on grounded theory is only 15.00. In addition, the score of student competency cultivation model based on grounded theory is 28.00, while the score of cultivation mechanism model based on content analysis method is 35.00. Therefore, the cultivation mechanism model established by the content analysis method can effectively make up for the deficiency of the model established based on the grounded theory, and students’ ability level of professional theory can be further improved.

### 4.4 Results discussion and analysis

Through the above experiments and data analysis, the following conclusions can be drawn. First, when comparing students’ ability identification, the ability training model based on grounded theory is compared with the ability training mechanism based on content analysis. The results show that with the increase of model iterations, the recognition accuracy of the ability model based on grounded theory and the ability training mechanism of content analysis method is continuously improved. However, the ability evaluation model based on grounded theory has higher accuracy, and the accuracy of ability evaluation even exceeds 78.52% when the number of iterations of the network model is 600. Secondly, through the statistics and comparison of the grading results of students of different majors, it is found that the ability training mechanism based on grounded theory can improve the overall ability level of students more significantly. Finally, by comparing the project scores under different training mechanisms, it is found that the ability training mechanism based on grounding theory performs better in reducing prediction error and scoring stability. Therefore, the ability training mechanism based on grounded theory can further improve students’ theoretical and cultural level. To sum up, the ability training mechanism based on grounded theory has shown good results in students’ ability identification, the improvement of professional theoretical knowledge and the stability of grading results, so it should be further popularized and applied to the ability training of tourism majors. This will help to enhance students’ employment competitiveness and personal development, and provide strong support for the sustainable development of tourism.

Tourism education is an important way to cultivate tourism talents, and how to improve the quality and competitiveness of tourism students has always been the focus of scholars’ attention. In this paper, a model of tourism undergraduate ability training mechanism based on grounded theory is constructed, and the model is evaluated by BP neural network. In order to better compare and verify the results of this paper, the relevant literature is also sorted out and analyzed. Phi et al. (2021) [42] introduced how learning based on design and value can cultivate innovative ability in tourism higher education. The research adopted mixed method, evaluated the effect of the course through questionnaire survey and focus group discussion among students who participate in the course, and put forward some suggestions for future course improvement. Lin et al. (2021) [43] discussed the cultural competence of Asian tourists. The research used questionnaire survey to collect data of tourists from different Asian countries, and analyzed the data of tourists’ openness, cultural identity and cultural IQ. The results show that Asian tourists show an open attitude of "mastering local culture and appreciating other cultures" in terms of cultural ability. Silitonga (2021) [44] introduced how competency-based education can improve the employment competitiveness of graduates from tourism vocational schools. The data of graduates from Indonesia Tourism Vocational School were collected by questionnaire, and the educational background and professional ability of graduates were analyzed. The results show that competency-based education can improve the employment competitiveness of graduates, especially if they have specific skills and knowledge in their fields. In addition, Kleib et al. (2023) [45] put forward an overview scheme on digital health education and training. The research adopted the method of scope review, aiming at sorting out and summarizing the related research on digital health education and training for undergraduate and graduate nursing students. Escribano et al. (2021) [46] evaluated the effectiveness of a standardized patient simulation training program for the training of chronic diseases and hospice care for undergraduate nursing students. Questionnaire survey and statistical analysis were used to confirm the effectiveness of the training program in improving the skills of nursing students. Archer-Kuhn et al. (2022) [47] discussed the methods of cultivating inquiry-based learning in social work education. The research results show that inquiry-based learning is of great significance in social work education. Liu et al. (2022) [48] put forward an evaluation method of undergraduate education innovation based on neural network and stress test. It was found that this evaluation method has positive significance for the improvement of undergraduate education. Bode (2022) [49] studied the cultivation of critical racial consciousness through visual culture and art education. The results showed that visual culture and art education played an important role in cultivating critical racial consciousness. Evans et al. (2023) [50] systematically reviewed the leadership training in undergraduate medical education. The research adopted the method of systematic review, summarized the relevant literature, and discussed the effect and influence of leadership training in undergraduate medical education. Hawker et al. (2022) [51] systematically reviewed the education and training of aseptic technique for undergraduate nursing students through mixed methods. The research comprehensively analyzed the results of questionnaire survey and qualitative research, and summarized the current situation and problems of aseptic technology education and training for undergraduate nursing students.

Based on the above literature research, the current research on the ability training mechanism of tourism students is diversified and involves different fields, which provides a useful reference for cultivating tourism talents with innovative ability and employment competitiveness. Among them, for the cultivation of innovative ability in tourism higher education, the method of learning based on design and value is put forward, and the course effect is evaluated through questionnaire survey and focus group discussion. The research on the cultural competence of Asian tourists points out that Asian tourists show an open attitude of "mastering local culture and appreciating other cultures" in their attitude towards culture. In addition, competency-based education also plays a positive role in improving graduates’ employment competitiveness, especially if they have specific skills and knowledge in the field.

## 5. Conclusion

With the rapid development of tourism, the demand for tourism professionals in the market is increasing. However, at present, undergraduate students majoring in tourism do not have enough theoretical knowledge, and their professional ability needs to be optimized. Therefore, on the basis of grounded theory, this paper analyzes the ability and training strategies of tourism undergraduate students, and optimizes the input parameters of the model by combining BP neural network. This paper analyzes the ability and training strategy of tourism undergraduates, and optimizes the ability evaluation model with BP neural network. The experimental results show that the recognition accuracy can reach over 78.52%, which provides support for the optimization and innovation of tourism undergraduates’ training strategy. In order to further improve the professional ability of undergraduate students majoring in tourism, it is suggested that schools strengthen curriculum, enrich educational resources, and guide students to actively participate in practical activities during their school days to improve their practical operation ability. In addition, people should do a good job in graduate employment guidance, carry out industry practice and enterprise internship, strengthen the connection between students and employers, and improve the quality and quantity of tourism talent supply. Therefore, this paper can provide support for the optimization and innovation of tourism undergraduate talent training strategy. Although this paper has achieved some experimental results by comparing the performance of different ability training mechanisms in the ability identification and scoring results of tourism majors, there are still some shortcomings. First, this paper only focuses on the ability training model based on grounded theory and content analysis, and does not involve other possible ability training methods, so how to choose the best training model remains to be further studied. Secondly, this paper only selects students majoring in tourism and music for comparison, and lacks the participation of students from other majors. Therefore, more empirical analysis is needed to determine whether the ability differences between students from different majors are universal. In addition, BP neural network is used as the evaluation model, but this model may have some limitations in dealing with nonlinear problems. Therefore, future research can consider introducing more machine learning algorithms or deep learning models to improve the accuracy and stability of ability identification and evaluation.

## References

[1] Budiman A, Nugraheni T, Purnomo P. The effect of architecture of arts education tourism towards interest in learning arts for high school students. Harmonia: Journal of Arts Research and Education, 2020, 20(2), pp.117–125.

[2] Astuti N N S, Ginaya G, Susyarini N P W A. Designing Bali tourism model through the implementation of tri hita karana and sad kertih values. International journal of linguistics, literature and culture, 2019, 5(1), pp. 12–23.

[3] Muhammad F J, Irawati R I, Halimah M. Policy Implementation of Sustainable Tourism Development Program in Manado City. Enrichment: Journal of Management, 2021, 12(1), pp. 1058–1070.

[4] Agasisti T, Egorov A, Zinchenko D, et al. Efficiency of regional higher education systems and regional economic short-run growth: empirical evidence from Russia[J]. Industry and innovation, 2021, 28(4), pp. 507–534.

[5] Walmsley A, Koens K, Milano C. Overtourism and employment outcomes for the tourism worker: impacts to labour markets[J]. Tourism Review, 2022, 77(1), pp. 1–15.

[6] Tosun C, Çalişkan C, Şahin S Z, et al. A critical perspective on tourism employment[J]. Current Issues in Tourism, 2023, 26(1), pp. 70–90.

[7] Finnerty S, Luke M, Duffy J T. A grounded theory of experiential group training of school counselors to engage in psychoeducational group lessons with first-in-family students. The Journal for Specialists in Group Work, 2019, 44(2), pp. 99–117.

[8] Claramita M, Setiawati E P, Kristina T N, et al. Community-based educational design for undergraduate medical education: a grounded theory study. BMC medical education, 2019, 19(1), pp. 1–10. 31296217 10.1186/s12909-019-1643-6 PMC6624922

[9] Prikhidko A, Su Y W, Houseknecht A, et al. Emotion regulation for counselors‐in‐training: A grounded theory. Counselor Education and Supervision, 2020, 59(2), pp. 96–111.

[10] Sinclair S, Hack T F, McClement S, et al. Healthcare providers perspectives on compassion training: a grounded theory study. BMC Medical Education, 2020, 20(1), pp. 1–13. doi: 10.1186/s12909-020-02164-8 32758216 PMC7403566

[11] Mousavi L, Kashef S M, Khodadadi M R, et al. Designing the Self-leadership Model of Elite Athletes (Based on Grounded theory Approach). Research on Educational Sport, 2021, 8(21), pp. 115–138.

[12] Hiltrop K, Heidkamp P, Breidenbach C, et al. Conflicting demands, coping, and adjustment: A grounded theory to understand rehabilitation processes in long‐term breast cancer survivors. Psycho‐Oncology, 2021, 30(11), pp. 1957–1964. doi: 10.1002/pon.5769 34272908

[13] Ahmady S, Khani H. The situational analysis of teaching-learning in clinical education in Iran: a postmodern grounded theory study. BMC medical education, 2022, 22(1), pp. 1–15. 35780110 10.1186/s12909-022-03577-3 PMC9250741

[14] Hilert A J, Haskins N. Teaching mindfulness in prison settings: a grounded theory of strategies to promote engagement and empowerment. Journal of Offender Rehabilitation, 2022, 61(1), pp. 1–19.

[15] Siroj K, Yorkulov M. Improving innovative training and national spiritualty for tourism education: Developing hospitality prospects in Uzbekistan. ACADEMICIA: AN INTERNATIONAL MULTIDISCIPLINARY RESEARCH JOURNAL, 2021, 11(1), pp. 1652–1656.

[16] Trong N P N, Phi N T N, Nguyen L T, et al. An assessment on impacts of online education on training quality and satisfaction of tourism undergraduate students in a private university and managerial implications for educators. International Research Journal of Management, IT and Social Sciences, 2021, 8(6), pp. 534–547.

[17] Dos A J E S. Didactic-pedagogical challenges in undergraduate courses in tourism in Brazil: Insights into teaching discourses. Современные проблемы сервиса и туризма, 2022, 16(2), pp. 41–56.

[18] Benuto L T, Casas J, O’Donohue W T. Training culturally competent psychologists: A systematic review of the training outcome literature. Training and Education in Professional Psychology, 2018, 12(3), pp. 125.

[19] Callahan J L, Watkins C E Jr. The science of training III: Supervision, competency, and internship training. Training and Education in Professional Psychology, 2018, 12(4), pp. 245.

[20] Cherdymova E I, Faleeva L V, Ilkevich T G, et al. Socio-Psychological Factors that Contribute to and Impede the Process of Student Eco-Vocational Consciousness Formation. Ekoloji, 2019, 28(107), pp. 133–140.

[21] Ruying L, Xiaozhen Z, Jianwen X. Based on the post competency as the core in the training mode of young teachers of human anatomy. Chinese Journal of Medical Education, 2020, 40(9), pp. 666.

[22] Chang X, Xue J. Research on the Evaluation and Promotion of Employees from the Perspective of Competency. Open Journal of Social Sciences, 2020, 8(02), pp. 99.

[23] Tran H H, Nguyen T H T, Hoang T T L, et al. The pedagogical training management of gifted high school teachers in the region of Hong river delta based on competency approach. JETT, 2021, 12(4), pp. 162–169.

[24] Dou Z, Sun Y, Zhu J, et al. The Evaluation Prediction System for Urban Advanced Manufacturing Development[J]. Systems, 2023, 11(8), pp. 392.

[25] Li Y Y, Au M L, Tong L K, et al. High-fidelity simulation in undergraduate nursing education: A meta-analysis[J]. Nurse education today, 2022, 111, pp. 105291. 35158134 10.1016/j.nedt.2022.105291

[26] Pong H K. The cultivation of university students’ spiritual wellbeing in holistic education: longitudinal mixed-methods study[J]. International Journal of Children’s Spirituality, 2021, 26(3), pp. 99–132.

[27] Barnes J, Rogerson C M. Student-centred VFR travel: Evidence from Johannesburg[J]. Urban Tourism in the Global South: South African Perspectives, 2021, pp. 173–191.

[28] Li J, Xue E. How talent cultivation contributes to creating world-class universities in China: A policy discourse analysis[J]. Educational Philosophy and Theory, 2022, 54(12), pp.2008–2017.

[29] Zhang L. Practical Teaching System Reform for the Cultivation of Applied Undergraduates in Local Colleges[J]. International Journal of Emerging Technologies in Learning (iJET), 2021, 16(19), pp.59–68.

[30] Du B, Chai Y, Huangfu W, et al. Undergraduate university education in internet of things engineering in china: A survey[J]. Education Sciences, 2021, 11(5), pp.202.

[31] Milawaty M, Andadari M, Aditya V, et al. Identification on the Need of English Public Speaking Class for Students at the Tourism Destination Study Program[J]. Journal of Language, Communication, and Tourism, 2023, 2(1), pp. 24–31.

[32] Ghebreyessus K, Ndip E M, Waddell M K, et al. Cultivating success through undergraduate research experience in a historically Black college and university[J]. Journal of chemical education, 2021, 99(1), pp. 307–316. 35979036 10.1021/acs.jchemed.1c00416 PMC9378306

[33] Kumar A, Ameh C. Start here-principles of effective undergraduate training[J]. Best Practice & Research Clinical Obstetrics & Gynaecology, 2022, 80, pp. 114–125. 34952793 10.1016/j.bpobgyn.2021.11.010

[34] Wang N, Rahman M N B A, Daud M A K B M. Diversified talent cultivation mechanism of early childhood physical education under the full-practice concept–oriented by preschooler mental health and intelligent teaching[J]. Frontiers in psychology, 2021, 11, pp. 593063. 33584429 10.3389/fpsyg.2020.593063 PMC7873970

[35] Scheibenzuber C, Hofer S, Nistor N. Designing for fake news literacy training: A problem-based undergraduate online-course[J]. Computers in Human Behavior, 2021, 121, pp. 106796. 36568041 10.1016/j.chb.2021.106796 PMC9761900

[36] Zhou Q. Research on the problems and countermeasures of the cultivation of adult college students’ innovation and entrepreneurship ability in the internet era[J]. Open Access Library Journal, 2021, 8(7), pp. 1–12.

[37] Gardner C J. Not teaching what we practice: Undergraduate conservation training at UK universities lacks interdisciplinarity[J]. Environmental Conservation, 2021, 48(1), pp. 65–70.

[38] Alkhaifi M, Clayton A, Kangasjarvi E, et al. Visual art-based training in undergraduate medical education: A systematic review[J]. Medical Teacher, 2022, 44(5), pp. 500–509. 34807802 10.1080/0142159X.2021.2004304

[39] Tomlinson M, Jackson D. Professional identity formation in contemporary higher education students. Studies in Higher Education, 2021, 46(4), pp. 885–900.

[40] Bakker A B, Hetland J, Olsen O K, et al. Daily strengths use and employee well‐being: The moderating role of personality. Journal of Occupational and Organizational Psychology, 2019, 92(1), pp. 144–168.

[41] Mínguez C, Martínez-Hernández C, Yubero C. Higher education and the sustainable tourism pedagogy: Are tourism students ready to lead change in the post pandemic era?[J]. Journal of Hospitality, Leisure, Sport & Tourism Education, 2021, 29, pp. 100329.

[42] Phi G T, Clausen H B. Fostering innovation competencies in tourism higher education via design-based and value-based learning[J]. Journal of Hospitality, Leisure, Sport & Tourism Education, 2021, 29, pp. 100298.

[43] Lin J H, Fan D X F, Tsaur S H, et al. Tourists’ cultural competence: A cosmopolitan perspective among Asian tourists[J]. Tourism Management, 2021, 83, pp. 104207.

[44] Silitonga P. Competency-based education: a multi-variable study of tourism vocational high school graduates[J]. Journal of Teaching in Travel & Tourism, 2021, 21(1), pp. 72–90.

[45] Kleib M, Arnaert A, Nagle L M, et al. Digital health education and training for undergraduate and graduate nursing students: a scoping review protocol[J]. JBI Evidence Synthesis, 2023, 21(7), pp. 1469–1476. 36728743 10.11124/JBIES-22-00266

[46] Escribano S, Cabañero-Martínez M J, Fernández-Alcántara M, et al. Efficacy of a standardised patient simulation programme for chronicity and end-of-life care training in undergraduate nursing students[J]. International Journal of Environmental Research and Public Health, 2021, 18(21), pp. 11673. 34770187 10.3390/ijerph182111673 PMC8583232

[47] Archer-Kuhn B, Lee Y, Hewson J, et al. Growing together: Cultivating inquiry-based learning in social work education[J]. Social Work Education, 2022, 41(3), pp.333–353.

[48] Liu C, Feng Y, Wang Y. An innovative evaluation method for undergraduate education: an approach based on BP neural network and stress testing[J]. Studies in Higher Education, 2022, 47(1), pp.212–228.

[49] Bode P. Visual culture art education to cultivate critical racial consciousness[J]. Art Education, 2022, 75(3), pp. 24–31.

[50] Evans M A, James E J, Mi M. Leadership Training in Undergraduate Medical Education: A Systematic Review[J]. International Journal of Medical Students, 2023, 11(1), pp. 58–66.

[51] Hawker C, Gould D, Courtenay M, et al. Undergraduate nursing students’ education and training in aseptic technique: A mixed methods systematic review[J]. Journal of Advanced Nursing, 2022, 78(1), pp. 63–77. 34258782 10.1111/jan.14974

